# Private Hospitals and Nursing Homes

**Published:** 1899-06-24

**Authors:** 


### Private Hospitals and Nursing Homes.

Much has been said lately about the "commissions" which, according to some people, medical men are in the habit of getting on all the various foods and drinks and instruments which they order for their patients, but so far as we can discover the whole thing is a mare's nest. Again we have heard a good deal about "dichotomy," or the division of the fee between consultants and the practitioners who recommend them —a practice which we are told is rampant at the present time in France, and has obtained a strong hold in the United States, and in regard to this also we have to thank Heaven that good old England still remains sufficiently conservative, and that the medical profession here is still recruited in sufficiently large degree from among gentlemen to render it difficult for all such forms of deception and false pretence to take root among us. Still the old slander keeps cropping up in one form or another, and now comes the complaint that eminent surgeons nowadays cannot do the simplest operation without sending their patient into a "home," and the suggestion that "of course" the lucky medico bags his percentage on the whole affair, the lodging, the board, the nursing, the dressings, and all the rest of it. We do not complain. We know too well that it is in human nature to impute evil motives, and for each man to measure his neighbour by himself. Still, when we meet with a wild accusation like this in the lay Press, it is not without interest to inquire whether the writer knows anything about the subject he descants upon; and we must confess that, after reading a long string of sneers levelled at "the doctors" in an article headed "The Private Hospital Deception" in one of the weekly papers, it was satisfactory to find the writer running up the flag of ignorance. He lays it down that "the most intricate of all operations requires only a case of instruments, a shilling's-worth of lint, a deal table, and a hospital nurse," and, that being his idea of the requirements of modern operative surgery, we need hardly be surprised at his wrath when he is "bundled off to a 'home'" even for "ordinary surgical treatment," especially as he asserts that a "ten guinea fee expands into a hundred guineas expenses of an illness," and all for the doctor's benefit!

Of course, this is absurd, but however well satisfied we may be that the writer of the article referred to does not understand the question, we must not too readily take for granted that even in folly truth does not lurk sometimes; and the truth in this matter is that surgery costs money which patients do not like to pay, and that if the best surgical results are to be obtained the "expenses" of operations must bear a larger proportion to the fee than was the case in the "shilling's-worth of lint" period. It is perfectly true that a few surgeons have private hospitals of their own, and that many have an interest, albeit not a monetary interest, in the success of the "homes" in which they do their operative work. But for whose advantage is all this? Surely it is for the benefit of the patients, purely and absolutely, that all this trouble is taken, although the surgeon does get the advantage in the form of fame and reputation and increase of practice, which must always attach to successful work, and is indeed its proper reward.

If it can be shown, as is suggested, that eminent surgeons name low fees, say of ten guineas, trusting to make their cases remunerative by means of "commission" out of the hundred guineas of "expenses" which will be incurred, then we agree that the system is a swindle and an abomination. But we absolutely deny that such things happen. What does happen is that a surgeon, knowing well that success in operative work hinges on a multitude of circumstances, and that his best efforts are liable to be rendered nugatory unless he has absolute control over all the surroundings of his patient, endeavours to give to his richer clients the same advantages which are at the disposal of his poorer ones in the public hospitals. He declines to make each operation an experiment by doing it amid unknown surroundings, and so he either runs a private hospital or obtains practical control over one or more nursing homes. Either hospital or home conducted under proper conditions must cost money, and this money the patients of course must pay. The sort of success which is now aimed at in high-class surgery is of a kind which would be quite unattainable under old conditions. The surgeon no longer feels fully armed so long as he has his "case of instruments" and his "shilling's-worth of lint." He no longer trusts to the chance assistance of a neighbouring general practitioner, or to the help of a nurse whom he has never seen before. He is now the central figure of a well-disciplined group, trained to work together, and accustomed to work amid certain surroundings as to the appropriateness of which he has satisfied himself by practical experience. And for all these things, and for their fitness for the purpose, the surgeon must hold himself responsible. So far are we from agreeing with those who declaim against "hotel-keeping doctors" and demand that the bill shall state how much they are paying for surgery and how much for soup, that we can have no doubt that the ideal plan would be that the surgeon should have his own hospital, his own nurses, his own everything, and while responsible for all, should accept an inclusive fee. That, however, cannot be, for many reasons, one great reason against it being the difficulty which such a system would place in the way of the younger men ever getting an opening. The present plan of surgical homes, each under the patronage and more or less direct control of eminent surgeons, works fairly well. It is expensive, no doubt, just as small hospitals are expensive, but we feel assured that the money paid goes to those who conduct these homes, who after all do not often roll in wealth, and that it certainly does not filter round, as is suggested, into the pockets of the doctors.

